# A Ribosomal Protein AgRPS3aE from Halophilic *Aspergillus glaucus* Confers Salt Tolerance in Heterologous Organisms

**DOI:** 10.3390/ijms16023058

**Published:** 2015-01-29

**Authors:** Xilong Liang, Yiling Liu, Lixia Xie, Xiaodan Liu, Yi Wei, Xiaoyang Zhou, Shihong Zhang

**Affiliations:** 1 College of Plant Sciences, Jilin University, Changchun 130062, China; E-Mails: xlliang09@mails.jlu.edu.cn (X.L.); liuyiling21th@126.com (Y.L.); lixiaxie82@126.com (L.X.); liuxiaodan1981@126.com (X.L.); wyziyu@163.com (Y.W.); yangxiaoZ897@163.com (X.Z.); 2 Agricultural College, Heilongjiang Bayi Agricultural University, Daqing 163319, China

**Keywords:** *Aspergillus glaucus*, ribosomal protein AgRPS3aE, salt tolerance, expression in hetero-organisms

## Abstract

High salt in soils is one of the abiotic stresses that significantly reduces crop yield, although saline lands are considered potential resources arable for agriculture. Currently, genetic engineering for enhancing salt tolerance is being tested as an efficient and viable strategy for crop improvement. We previously characterized a large subunit of the ribosomal protein RPL44, which is involved in osmotic stress in the extremely halophilic fungus *Aspergillus glaucus*. Here, we screened another ribosomal protein (AgRPS3aE) that also produced high-salt tolerance in yeast. Bioinformatics analysis indicated that *AgRPS3aE* encodes a 29.2 kDa small subunit of a ribosomal protein belonging to the RPS3Ae family in eukaryotes. To further confirm its protective function against salinity, we expressed *AgRPS3aE* in three heterologous systems, the filamentous fungus *Magnaporthe oryzae* and two model plants *Arabidopsis* and tobacco. Overexpression of *AgRPS3aE* in all tested transformants significantly alleviated stress symptoms compared with controls, suggesting that *AgRPS3aE* functions not only in fungi but also in plants. Considering that ribosomal proteins are housekeeping components in organisms from prokaryotes to eukaryotes, we propose that *AgRPS3aE* is one of the optimal genes for improving high-salt tolerance in crops.

## 1. Introduction

Soil salinization is an increasingly serious environmental problem on a global scale. It is estimated that 6.5% of the total land has been salinized, and this area continues to expand [1,2]. High salt in soils imposes multiple negative effects on plant growth and development and thus reduces crop yield. To alleviate or overcome salt stress, plants have developed several resistance mechanisms, including the regulation of ion homeostasis and the activation of certain metabolic components [3,4]. However, if the salinity is beyond a plant’s ability to tolerate, physiological damage will occur, followed by homeostatic disruption and ion toxicity, and even leading to the death of plants [5]. Most cultivar crops, such as cabbage, radish, rice and soybean, are sensitive to high-salt stress [6]. Therefore, to use saline soils for planting and to increase crop production, it is crucial to enhance the salt tolerance of plants. Traditional breeding is limited by the long duration of breeding and incompatibility of distant-hybridization and thus has limited success in efficiently improving the salt tolerance of crops [7]; therefore, genetic engineering via the transfer of salt-tolerance genes remains the most promising option [8].

Ribosomal proteins, as primary components of ribosomes, are mainly responsible for protein synthesis in cells. In recent years, extra-ribosomal functions of these proteins have gained much attention, leading to increased interest in studying the genes encoding ribosomal proteins [9,10]. RPS3aE, also called RPS1 in yeast or RPS3a in other eukaryotes, is located in the 40S small subunit of ribosomes [11,12]. Many studies have indicated that RPS3aE is involved in a number of cellular processes, including protein synthesis, cell growth, apoptosis and tumorigenesis [13,14,15,16,17]. Interestingly, RPS3a has also been reported to alleviate copper stress in *Argopecten purpuratus* [18]. In another study, soybean RPS3a was shown to be associated with both flooding tolerance and disease resistance to *Phytophthora sojae* [19], suggesting that RPS3a is a multitasking protein that plays extra-ribosomal roles in stress tolerance.

In a previous study [20], *Aspergillus glaucus*, an extremely halophilic fungus that can survive in a saturated sodium chloride (32%, *m*/*v*) environment, was isolated by our laboratory. Using total mRNA of *A. glaucus*, a full-length cDNA yeast expression library was constructed [20]. Based on this system, we screened a series of yeast colonies harboring individual salt-tolerance genes from *A. glaucus*, including the previously studied large subunit of ribosomal protein (AgRPL44), which enhances osmotic tolerance in tobacco [20]. In the present work, another ribosomal protein, the small subunit AgRPS3aE, was isolated through screening the same yeast expression cDNA library. The evolution and primary structure of AgRPS3aE were assessed using bioinformatics analysis, and the salt tolerance was biologically analyzed in *Magnaporthe oryzae*, *Arabidopsis thaliana* and tobacco. To our knowledge, RPS3aE has not previously been reported to be involved in salt tolerance prior to this research.

## 2. Results

### 2.1. AgRPS3aE Gene and Protein Sequences from A. glaucus

**Figure 1:**
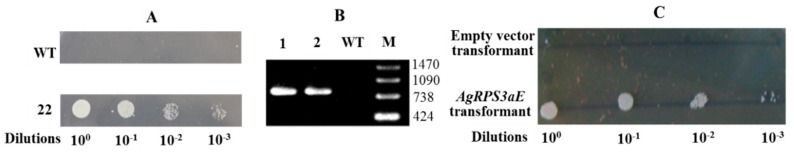
Cloning of the *AgRPS3aE* gene and verification of salt tolerance. (**A**) A salt-tolerant transformant was screened from a yeast cDNA expression library on solid SD minimal medium (−uracil, +galactose) containing 20% (*w*/*v*) NaCl for 3–5 days at 30 °C; (**B**) The *AgRPS3aE* gene was amplified using a salt-tolerant colony as a template. Lane 1, Lane 2: *AgRPS3aE* gene; Lane M: Marker; and (**C**) The salt-tolerance properties conferred by the *AgRPS3aE* gene were verified by the heterologous expression of *AgRPS3aE* in yeast strain AH109. The empty vector transformant could not survive in a high-salt environment. The transformants were cultivated under 20% NaCl for 3–5 days at 30 °C.

To isolate salt-tolerance genes, we screened the yeast expression library containing cDNA for *A. glaucus* using SD minimal medium (0.67% yeast nitrogen base, 2.0% galactose, 2.0% agar, -Ura) supplemented with 20% NaCl, and obtained a colony that was able to grow on salt plates (Figure 1A). PCR amplification (Figure 1B) and DNA sequencing revealed that a cDNA fragment that contains an ORF of 762 bp (*AgRPS3aE*) was inserted into the expression vector pYES2-DEST52. Bioinformatics analysis indicated that the ORF encodes a 40S ribosomal protein (AgRPS3aE) of 254 amino acids with a predicted molecular mass of 29.2 kDa and a theoretical pI of 10.21. When the cDNA was subcloned into the vector pRUL129 and re-transformed into the wild-type yeast AH109, we obtained the same salt-tolerant result (Figure 1C).

### 2.2. Evolution and Primary Structure of the Ribosomal Protein AgRPS3aE

**Figure 2:**
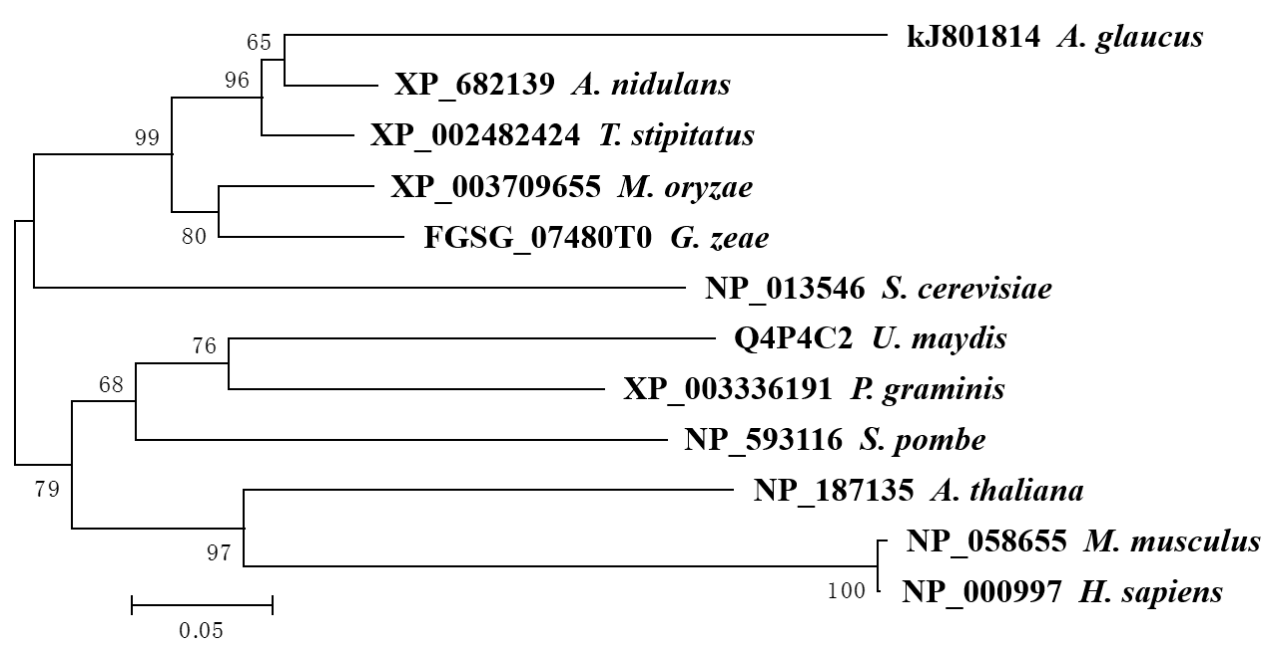
Phylogenetic relationships of AgRPS3aE and RPS3aE from various representative species. A neighbor-jointing tree was constructed with MEGA5.2. Bootstrap values are shown as percentages from 500 replications at branch points. The estimated genetic distance between sequences is proportional to the lengths of the horizontal lines connecting one sequence to another. The GenBank accession numbers of all RPS3aE genes are followed by their species names. *A. glaucus*: *Aspergillus glaucus*, *A. nidulans*: *Aspergillus nidulans*, *T. stipitatus*: *Talaromyces stipitatus*, *M. oryzae*: *Magnaporthe oryzae*, *G. zeae*: *Gibberella zeae*, *S. cerevisiae*: *Saccharomyces cerevisiae*, *U. maydis*: *Ustilago maydis*, *P. graminis*: *Puccinia graminis*, *S. pombe*: *Schizosaccharomyces pombe*, *A. thaliana*: *Arabidopsis thaliana*, *H. sapiens*: *Homo sapiens*, *M. musculus*: *Mus musculus*.

**Figure 3:**
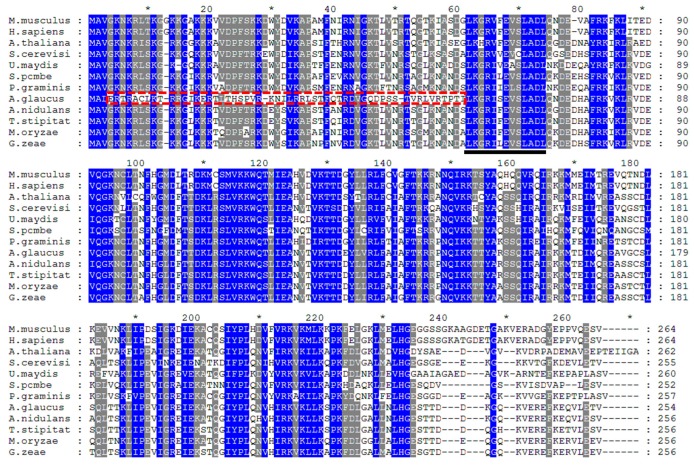
Multiple alignment of AgRPS3aE with RPS3aE from other species in GenBank. Identical residues are shown in blue, and similar residues are shown in grey. The RPS3aE signature motif is underlined. The amino acid sequence indicated by the dotted line is unique to AgRPS3aE. * represents the amino acid number increased by twenty from the 10th amino acid.

To further characterize AgRPS3aE, 12 RPS3aE homologues from different species were aligned. A phylogenetic tree was constructed using the neighbor-joining method. In the phylogenetic tree (Figure 2), AgRPS3aE and other homologues from five Ascomycota fungi were in the same large subgroup, indicating a close revolutionary relationship among these proteins. Alignment of amino acid sequences revealed that AgRPS3aE shared high similarities (78.35%, 78.35%, 77.56%, 72.05% and 58.50%, respectively) with RPS3aE from *A. nidulans*, *T. stipitatus*, *M. oryzae*, *G. zeae* and *S. cerevisiae* (Figure 3; Table S1). In addition, the ribosomal protein S3aE signature motif [21] was also recognized in AgRPS3aE (LK[G/H]R[I/V]XEX[S/C]LADL) using Motif Scan (http://hits.isb-sib.ch/cgi-bin/PFSCAN). Interestingly, the AgRPS3aE sequence is somewhat variable in the *N*-terminal region (4–61 aa) when compared with the RPS3aE sequences of other species, including two protein kinase C phosphorylation sites (12–14, 49–51 aa), one cAMP- and cGMP-dependent protein kinase phosphorylation site (32–35 aa) and one *N*-myristoylation site (43–48 aa). These unique features in the protein sequence likely confer the extra-ribosomal protein functions of AgRPS3aE.

### 2.3. AgRPS3aE Confers Tolerance to Osmotic Stress in M. oryzae

**Figure 4:**
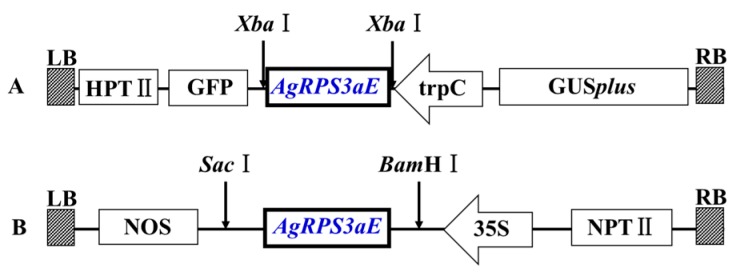
Construction of vectors containing *AgRPS3aE* for transformation. (**A**) T-DNA map of the binary vector pGFPGUS*plus*-trpC::*AgRPS3aE* used to transform *M. oryzae*; (**B**) T-DNA map of the binary vector pBI121::*AgRPS3aE* used for the *Arabidopsis* and tobacco transformations. GFP: green fluorescent protein gene; HPTII: hygromycin resistance gene; GUS*plus*: β-glucuronidase gene; trpC: *A. nidulans* TrpC promoter; 35S: CaMV 35S promoter; NOS: NOS terminator; NPTII: kanamycin resistance gene.

**Figure 5:**
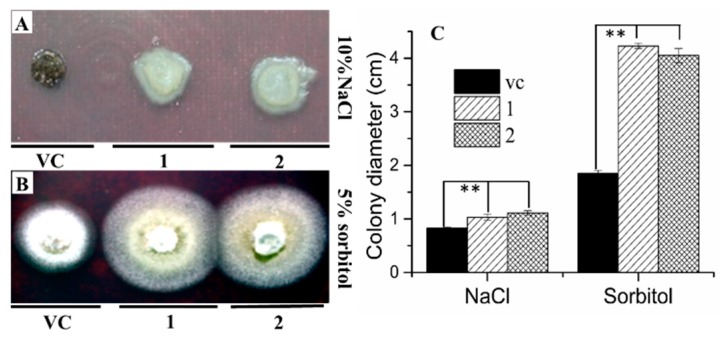
Assay for osmotic stress resistance in *AgRPS3aE*-transformed *M. oryzae*. The transformants were incubated on CM containing 10% NaCl (**A**) or 5% sorbitol (**B**) for seven days at 28 °C; (**C**) The response bar graph for colony diameter was measured to illustrate the osmotic stress resistance of *AgRPS3aE*-transformed *M. oryzae*. The numbers 1 and 2 represent two different transgenic strains. ** indicates a significant difference at *p* < 0.01.

The blast fungus *M. oryzae* was employed to confirm the protective function of *AgRPS3aE* under osmotic stress (NaCl and sorbitol) due to its sensitivity to salt and convenience for transformation. We constructed an overexpression vector of *AgRPS3aE* (pGFPGUS*plus*-trpC::AgRPS3aE) (Figure 4A). Using the ATMT method, the recombinant *AgRPS3aE* was transferred into *M. oryzae*. Transgenic candidates were confirmed using PCR and RT-PCR. *Magnaporthe* transformants were inoculated on solid CM medium (10 g/L glucose, 2 g/L peptone, 1 g/L yeast extract, 1 g/L casamino acids, 0.1% (*v*/*v*) trace elements, 0.1% (*v*/*v*) vitamin supplement, 6 g/L NaNO_3_, 0.5 g/L KCl, 0.5 g/L MgSO_4_, 1.5 g/L KH_2_PO_4_, pH 6.5, 20 g/L agar) supplemented with 10% NaCl or 5% sorbitol. Transformants expressing AgRPS3aE grew better under osmotic stress than did the controls (Figure 5A,B). The mycelial colony diameters of the *AgRPS3aE* transformants were 1.24- and 1.33-fold larger after growth on 10% NaCl solid CM than those of the transformants containing empty vector. Compared with the growth of the *AgRPS3aE* transformants, the radial growth of the transformants containing empty vector on solid CM medium supplemented with 5% sorbitol was reduced by 56.25% and 54.35%, respectively (Figure 5C). These results further confirmed that the *AgRPS3aE* gene was important in improving resistance to osmotic stress.

### 2.4. Overexpression of AgRPS3aE Enhances Salt Tolerance of Plants

**Figure 6:**
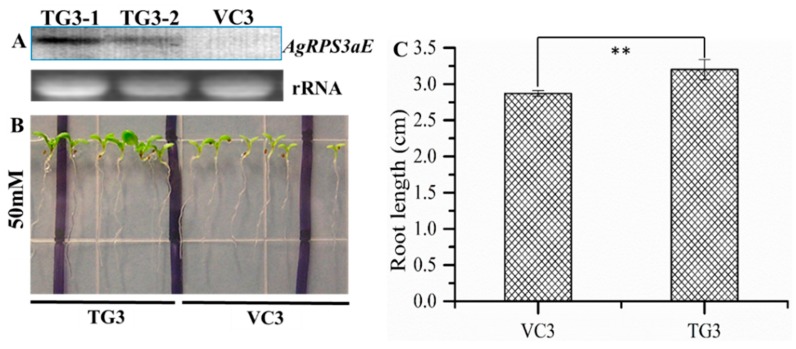
Effect of *AgRPS3aE* overexpression on salt tolerance in transgenic *Arabidopsis*. (**A**) Northern blotting analysis of three independent T3 generation transgenic *Arabidopsis* lines; (**B**) The performance comparison of T3 and VC3 *Arabidopsis* seedlings stressed by 50 mM NaCl for 15 days; (**C**) The response bar graph for root length was calculated to illustrate the salt tolerance of *AgRPS3aE* transgenic *Arabidopsis*. The data presented are the means ± SD of three replications. ** indicates a significant difference at *p* < 0.01.

**Figure 7:**
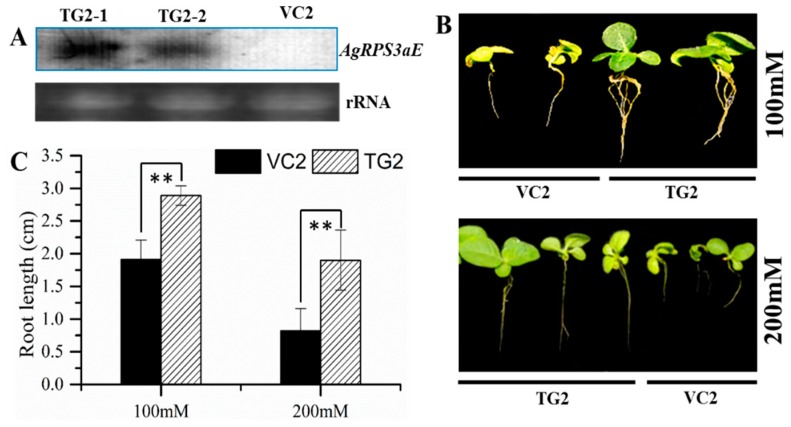
Effect of salt stress on tobacco seedlings from VC2 and T2 progenies of transgenic lines overexpressing *AgRPS3aE*. (**A**) Northern blotting analysis of three independent T2-generation transgenic tobacco lines; (**B**) The phenotypes of VC2 and T2 progenies tobacco seedlings stressed with 100 mM or 200 mM NaCl for 15 days; (**C**) The root lengths of VC2 and T2 progenies tobacco seedlings stressed with 100 or 200 mM NaCl for 15 days. The data are presented as the means ± SD of three replications. Significant differences of the VC2 and T2 progenies root lengths in tobacco seedlings are indicated as ** *p* < 0.01.

Because the *AgRPS3aE* gene increased the osmotic stress tolerance of *A. glaucus* and *M. oryzae*, transgenic *Arabidopsis* and tobacco plants containing *AgRPS3aE* were generated based on the binary vector pBI121::*AgRPS3aE* (Figure 4B) to evaluate whether the overexpression of this gene could enhance plant salt tolerance. Kanamycin-resistant transgenic lines were confirmed by Northern blotting (Figure 6A and Figure 7A). When the transgenic plants (TG) harboring *AgRPS3aE* and the corresponding vector-control plants (VC) were exposed to NaCl stress, the TG plants all appeared much healthier than the control *Arabidopsis* plants (VC3) or tobacco seedlings (VC2), especially in terms of leaf area and root length. For example, after treatment with 50 mM NaCl for 15 days, the root length of the TG3 lines of *Arabidopsis* was 3.20 ± 0.14 cm, whereas the root length of the VC3 lines was only 2.87 ± 0.04 cm (Figure 6B,C, *p* < 0.01). Similar results were also found for transgenic tobacco seedlings (Figure 7B,C, *p* < 0.01). As the NaCl concentration increased, the inhibition of root elongation was markedly reduced in transgenic tobacco plants (TG2) compared with the control tobacco plants (VC2). At NaCl concentrations of 100 and 200 mM, the root lengths of the VC2 lines were 33.91% (*p* < 0.01) and 43.16% (*p* < 0.01) lower than those of the TG2 lines. However, the TG3 *Arabidopsis* and TG2 tobacco were randomly selected for planting under salt-free conditions. No evident morphological differences were observed between the TG and corresponding VC seedlings (data not shown). These results demonstrated that the transgenic plant seedlings overexpressing *AgRPS3aE* were significantly more halotolerant than the wild-type seedlings.

## 3. Discussion

Identifying salt-tolerant genes is one of the critical steps for biological management of saline sodic lands [22]. In this report, a novel ribosomal protein gene associated with salt tolerance was isolated from the extremophile *A. glaucus* using a cDNA yeast expression library. Sequence analysis revealed that this gene encodes a ribosomal protein belonging to the S3Ae family in eukaryotes, and the gene was therefore named *AgRPS3aE*. Ribosomal proteins, the major components of the ribosome complex, are generally considered to be involved in protein translation. However, the extra-ribosomal functions of these proteins as independent polypeptides have recently begun to receive attention and require further study [23].

Previous studies have shown that many ribosomal protein (RP) genes are involved in the response of plants to environmental stresses. For example, transcription of *BnC24* (also named *BnRPL13*), a cold-regulated gene, can be induced by cold stress in *Brassica napus* [24]. Transcript levels of *GmRPS13*, *GmRPS6* and *GmRPL37* dramatically increase after soybeans are exposed to cold temperatures [25]. In maize, the expression of *RPL10* genes is induced by UV-B radiation, whereas in *Arabidopsis*, these genes are differentially regulated by this type of radiation [26]. In addition, a recent analysis of the differential expression of RP genes in *Arabidopsis* roots upon phosphate or iron deficiency revealed that 579 RP genes are involved in abiotic stress responses, including the response to salt stress [13]. Therefore, some ribosomal proteins are important for protection against multiple abiotic stresses in plants.

The *RPS3aE* gene has also been reported to be involved in stress responses such as copper stress [18], flood stress, *Phytophthora sojae* resistance [19], and arsenite stress [27]. However, few reports have described the role of *RPS3aE* gene overexpression in salt tolerance. To characterize the role of *AgRPS3aE* in salt tolerance, recombinant species containing *AgRPS3aE* were constructed. The salt-tolerant properties of *AgRPS3aE* were demonstrated in *M. oryzae* and two model plant species, *Arabidopsis* and tobacco. These observations provide strong evidence that *AgRPS3aE* confers salt tolerance not only in fungi but also in plants.

Many studies have shown that ribosomal protein genes, including *RPS3a*, are highly conserved housekeeping genes [28,29,30]. In addition, amino acid alignment revealed that the AgRPS3aE protein from *A. glaucus* shared high degrees of similarity with orthologues in both mammals and plants. Thus, S3Ae proteins among different organisms possess many similar characteristics. Negative effects such as possible environmental and food risks from gene transfer across species will also be decreased. Therefore, the increased salt tolerance conferred by *AgRPS3aE* in plants makes it a potential bioresource for breeding salt-tolerant crops.

Although RPS3aE is highly conserved in many organisms, the finding that *AgRPS3aE* conferred salt tolerance to *M. oryzae*, *Arabidopsis* and tobacco demonstrated that AgRPS3aE had a distinctive function. Moreover, multiple sequence alignment also revealed that the *N*-terminal region (4–61 aa) of AgRPS3aE protein contains certain specific amino acid sequences that differ from those of other RPS3aE proteins. These amino acid sequences specific to AgRPS3aE including important functional sites, such as protein kinase C phosphorylation sites (12–14 aa TRR, 49–51 aa TVR), a cAMP- and cGMP-dependent protein kinase phosphorylation site (32–35 aa RRLS) and an N-myristoylation site (43–48 aa VGQDPS) (T-Thr; R-Arg; V-Val; L-Leu; S-Ser; G-Gly; Q-Gln; D-Asp; P-Pro), which were identified using a database of protein domains, families and functional sites (http://prosite.expasy.org/prosite.html). Previous studies have indicated that these sites can enhance or inhibit the function of the protein by combining with corresponding activator or inhibitor proteins [31]. For example, AgRPS3aE has sites that are known to be involved in membrane receptor-mediated signal transduction, suggesting that AgRPS3aE is involved in a variety of signal transduction pathways [32,33]. Together, these results indicate that the unique amino acid sequences of the *N*-terminal region (4–61 aa) may play very important roles in the remarkable resistance to salt stress conferred by the *AgRPS3aE* gene. Therefore, it is crucial to perform site-specific mutagenesis to test this hypothesis in the future.

## 4. Experimental Section

### 4.1. Organisms and Growth Conditions

In this study, *A. glaucus* (10^6^–10^10^ spores/mL) isolated from air-dried wild vegetation at the surface periphery of a solar salt field was incubated in potato dextrose broth containing 10% NaCl and cultured for approximately six days at 30 °C. For *Escherichia coli* strain DH5α (Sangon, Shanghai, China), standard procedures were used to manipulate the bacterial cells and recombinant DNA [34]. *Saccharomyces cerevisiae* strains used in the study were His-, Leu-, Trp-, and Ura-INVSc1 (Invitrogen, Carlsbad, CA, USA) and AH109 (Takara, Dalian, China). Yeast cells were grown in SD minimal medium (−uracil, +galactose, FunGenome Co., Ltd., Beijing, China) or yeast potato dextrose (YPD; 2% peptone, 1% yeast extract, and 2% glucose), and the cultures were maintained by plating on YNB-glucose medium supplemented with 2% (*w*/*v*) agar. *Agrobacterium tumefaciens* (LBA4404), *Arabidopsis* (Columbia-0) and tobacco (*Nicotiana tabacum* cv. SR-1) all came from our laboratory. The plants for transgenosis were grown at 22 °C in a 12-h light/12-h dark cycle in individual pots (diameter 12 cm, depth 12 cm) on soil (GS 90 soil mixed with vermiculite in a ratio 2:1 (*v*/*v*)).

### 4.2. Cloning and Sequence Analysis of the AgRPS3aE Gene

In previous studies, a yeast expression library containing cDNA prepared from *A. glaucus* treated with 5%, 10%, and 20% (*w*/*v*) NaCl was constructed and stored in our lab [20]. Screening of the yeast expression library was performed on SD minimal medium supplemented with 20% NaCl. Several yeast transformants that were able to tolerate 20% NaCl were obtained. The inserted cDNA fragments were identified via PCR amplification using the primers 5'-GACTGGTTCCAATTGACAAGC-3' and 5'-GCAAATGGCATTCTGACATCC-3'. The purified products were cloned into the pMD-18T vector (Takara, Dalian, China) and sequenced (Sangon Biotech, Shanghai, China). Subsequently, the molecular weight (*M*w) and isoelectronic point (pI) predictions for the deduced AgRPS3aE protein were performed using the ProtParam tool (http://www.expasy.org/tools/protparam.html). A phylogenetic tree was constructed with MEGA5.2. A multiple sequence alignment was performed using the alignment programs Clustal Omega (http://www.ebi.ac.uk/Tools/msa/clustalo/) and GeneDoc 3.2. Important functional sites were identified using a database of protein domains, families and functional sites (http://prosite.expasy.org/prosite.html). To increase the accuracy, these determined sites needed to meet the following criteria: (1) horizontal scaling higher than or equal to 0.6 and (2) twenty hits on one sequence by five distinct patterns. The *AgRPS3aE* open reading frame (ORF) was amplified from the clone by thirty cycles of PCR (98 °C for 10 s; 53 °C for 15 s; 72 °C for 1 min) with the primers 5'-CGGGATCCATGGCGTTGGAAAGAACAAGG-3' (forward, *Bam*HI site underlined) and 5'-CGGGATCCTCAGACGGTCTCAAGAACCTGTT-3' (reverse, *Bam*HI site underlined). The PCR was performed in a 25 μL containing 0.2 μg plasmid template, 2.5 μM primers, 2.5 mM dNTP mixture, 0.75 U of PrimeSTAR HS DNA Polymerase (Takara, Dalian, China), and the reaction buffer. The PCR products were digested with *Bam*HI and directionally cloned into the vector pRUL129 (preserved in our lab). The pRUL129 vector and recombinant plasmid pRUL129::*AgRPS3aE* were transformed into strain AH109. The transformants were selected and cultured in synthetic medium (yeast nitrogen base, 2% glucose) lacking uracil. Colonies that were positive for the pRUL129::*AgRPS3aE* plasmid were further confirmed based on their NaCl tolerance.

### 4.3. Analysis of Osmotic Stress-Tolerance in M. oryzae Containing AgRPS3aE

The model fungus *M. oryzae* is sensitive to salt stress and is convenient for gene transformation. Therefore, *M. oryzae* was used to quickly verify the osmotic stress tolerance of *AgRPS3aE*. The *AgRPS3aE* gene was digested using *Xba*I and inserted into the *Xba*I site of the pGFPGUS*Plus*-trpC vector (reconstructed on the basis of pGFPGUS*plus*). The reconstructed expression vector was introduced into *Agrobacterium tumefaciens* AGL-1 from our laboratory by a heat-shock method and then transformed into the *M. oryzae* 70–15 strain stored in our laboratory using the method of *Agrobacterium tumefaciens*-mediated transformation (ATMT) as previously described [35]. Candidate transformants were selected with 250 µg/mL hygromycin B (Roche, Mannheim, Germany) and were identified using PCR and RT-PCR. Conditions of the PCR and RT-PCR (containing 2.5 U Taq DNA polymerase and the reaction buffer, 2.5 μM primers, 2.5 mM dNTP mixture, 0.1 μg template in a 25 μL) are as follows: 94 °C 4 min, then 30 cycles at 94 °C 30 s, 56 °C 30 s, 72 °C 1 min. Subsequently, the four transformants were purified using single conidia isolation, and a representative transformant was selected for the next test.

The mycelia blocks (0.8 cm in diameter) of *M. oryzae*, which were obtained from the edge of a complete minimal medium (CM) plate, were inoculated on solid CM containing 10% NaCl or 5% sorbitol and cultured 7–10 days at 28 °C. Vegetative growth and colony diameter were determined and photographed to investigate the osmotic stress tolerance.

### 4.4. Northern Blotting Analysis

Total RNA was extracted using TRIzol reagent (Invitrogen, Carlsbad, CA, USA). Then, 10 μg of total RNA from each sample of *Arabidopsis* and tobacco was transferred onto nylon film via high-salt (20× SSC: 3 M NaCl, 0.3 M sodium citrate, pH 7.0) solution after electrophoresis in 1% agarose gel and denatured with 6% formaldehyde [36]. Northern blotting was performed using full-length *AgRPS3aE* cDNA labeled with DIG-dUTP as a probe according to the protocol provided with the DIG-High Prime DNA Labeling and Detection Starter Kit I (Roche, Mannheim, Germany) [37].

### 4.5. Plant Transformation and Salt Tolerance Analysis

The *AgRPS3aE* ORF was amplified from the *A. glaucus* cDNA clone via PCR using the primers 5'-CGGGATCCATGGCGTTGGAAAGAACAAGG-3' (forward, *Bam*HI site underlined) and 5'-CGAGCTCTCAGACGGTCTCAAGAACCTGTT-3' (reverse, *Sac*I site underlined). The amplified products were digested with *Bam*HI (Takara, Dalian, China) and *Sac*I (Takara, Dalian, China) and directionally cloned into the plant expression vector pBI121 (Ding-guo, Beijing, China) containing the CaMV 35S promoter, NOS terminator and NPTII (kanamycin) selection marker. The recombinant plasmid pBI121::*AgRPS3aE* was introduced into *A. tumefaciens* (LBA4404) for *Arabidopsis* (Columbia-0) transformation using the floral dip method [38] and for tobacco (*Nicotiana tabacum* cv. SR-1) transformation by *Agrobacterium*-mediated leaf disc infiltration [39]. *AgRPS3aE* transgenic seedlings were selected on Murashige and Skoog medium with a final concentration of 40 mg/L kanamycin. PCR and Northern blotting were used for further confirmation. T3 generation *Arabidopsis* (TG3), T2 generation tobacco (TG2), and the empty pBI121 vector control lines (VC3 or VC2) were used for biological assays.

For the salt tolerance assay, 15-day-old seedlings of TG3 and VC3 *Arabidopsis* were examined and photographed in 1/2 MS medium supplemented with 50 mM NaCl. The three-week-old TG2 and VC2 tobacco seedlings were placed in Hoagland’s nutrient solution containing NaCl at different concentrations (100 and 200 mM). These tobacco seedlings were then examined and photographed after they were cultured vertically for 15 days.

## 5. Conclusions

This study presents the screening of the novel salt-tolerance gene *AgRPS3aE*, which encodes a 29.2 kDa small subunit of a ribosomal protein. The overexpression of *AgRPS3aE* in three heterologous systems (*M. oryzae*, *Arabidopsis*, and tobacco) confirms its protective function against salinity, demonstrating that *AgRPS3aE* functions not only in fungi but also in plants. Considering the housekeeping functions of ribosomal proteins in both prokaryotes and eukaryotes, we propose that *AgRPS3aE* is one of the optimal genes for improving crop tolerance to high-salt conditions.

## Supplementary Materials

Supplementary materials can be found at http://www.mdpi.com/1422-0067/16/02/3058/s1.
